# Rhyme Awareness in Children With Normal Hearing and Children With Cochlear Implants: An Exploratory Study

**DOI:** 10.3389/fpsyg.2019.02072

**Published:** 2019-09-12

**Authors:** Linye Jing, Katrien Vermeire, Andrea Mangino, Christina Reuterskiöld

**Affiliations:** ^1^Department of Communicative Sciences and Disorders, New York University, New York, NY, United States; ^2^Department of Communication Sciences and Disorders, Long Island University, Brooklyn, NY, United States; ^3^LIJ Hearing and Speech Center, Cohen Children’s Medical Center, Northwell Health, New Hyde Park, NY, United States

**Keywords:** rhyme awareness, neighborhood density, cochlear implants, vocabulary, working memory

## Abstract

Phonological awareness is a critical component of phonological processing that predicts children’s literacy outcomes. Phonological awareness skills enable children to think about the sound structure of words and facilitates decoding and the analysis of words during spelling. Past research has shown that children’s vocabulary knowledge and working memory capacity are associated with their phonological awareness skills. Linguistic characteristics of words, such as phonological neighborhood density and orthography congruency have also been found to influence children’s performance in phonological awareness tasks. Literacy is a difficult area for deaf and hard of hearing children, who have poor phonological awareness skills. Although cochlear implantation (CI) has been found to improve these children’s speech and language outcomes, limited research has investigated phonological awareness in children with CI. Rhyme awareness is the first level of phonological awareness to develop in children with normal hearing (NH). The current study investigates whether rhyme awareness in children with NH (*n* = 15, median age = 5; 5, IQR = 11 ms) and a small group of children with CI (*n* = 6, median age = 6; 11.5, IQR = 3.75 ms) is associated with individual differences in vocabulary and working memory. Using a rhyme oddity task, well-controlled for perceptual similarity, we also explored whether children’s performance was associated with linguistic characteristics of the task items (e.g., rhyme neighborhood density, orthographic congruency). Results indicate that there is an association between vocabulary and working memory and performance in a rhyme awareness task in NH children. Only working memory was correlated with rhyme awareness performance in CI children. Linguistic characteristics of the task items, on the other hand, were not found to be associated with success. Implications of the results and future directions are discussed.

## Introduction

Successful literacy learning is the most important task for children to achieve in school. Seminal work as Liberman (1973), Lundberg et al. (1988) has shown that phonological awareness skills, a critical component of phonological processing, are closely linked to children’s literacy outcomes. Phonological awareness enables children to actively analyze and reflect upon the sound structure of words. It facilitates the sound-to-letter knowledge required for decoding (i.e., reading) and encoding (i.e., spelling). To master reading and writing, children need to learn to decode written words. This decoding ability is highly dependent on phonological awareness skills, which enable children to break down speech into smaller phonological units such as words, syllables, onsets and rimes, and phonemes (see Torgesen et al., 1994; Adams, 1998).

Different tasks have been used to assess children’s phonological awareness skills. In segmentation tasks, children break down a whole word into smaller phonological units by clapping out the number of syllables or sounds in a word. In identification tasks, children distinguish specific sounds within a word (e.g., Is there a/s/in “Mom”?). In manipulation tasks, children delete or substitute smaller units within a word (e.g., What is left if you take/um/away from “umbrella”?). Children are commonly asked to participate in such listening tasks during their early school years, and these tasks are included in phonological awareness tests. Strong performance in these tasks entail both sharp listening skills, as well as metalinguistic skills (i.e., making judgments about the linguistic structure of the items).

In this paper, we explore the potential relationship between different levels of hearing experience, vocabulary skills, and non-verbal working memory skills on success in a rhyme recognition task in a group of children, which includes a small group of children with cochlear implants (CI). All children with CI were congenitally deaf and implanted before the age of two. A carefully designed rhyme recognition task with a balanced rhyme density neighborhood, orthographic congruency, and the type of phoneme substitutions of the items, as well as a tight control for the perceptual saliency of phonemes, age of acquisition, and familiarity of the stimuli words, was used. This allowed us to explore how linguistic factors might be associated with accuracy in a task measuring rhyme awareness.

### Development of Phonological Awareness in Children

There is a consensus that the grain size of phonological representation (i.e., syllable, onset/rime and phoneme) in typically developing (TD) children develops from larger to smaller units (Ziegler and Goswami, 2005). Onset-rime awareness is the first to appear at around age four, as shown in a seminal study by Bradley and Bryant (1983). Children were asked to identify the odd word from three to four single-syllable words with CVC (i.e., consonant – vowel – consonant) structure. The odd word differs from the rest by not sharing the same initial (e.g., *b*us, *b*un, *r*ug), medial (e.g., p*i*n, b*u*n, g*u*n) and final (e.g., do*ll*, ho*p*, to*p*) phonemes. Results showed that the shared consonants in the initial positions (i.e., onset) as well as the combination of medial vowels and final consonants (i.e., rime) are the basis for making correct judgments in the oddity tasks. Four- and five-year-old children performed above chance level in both the onset and rime versions of the oddity task, suggesting proficiency in rhyme awareness (Bradley and Bryant, 1983; Kirtley et al., 1989). In other studies, children were asked to identify pairs of rhyming words instead of the odd word or the non-rhyming word (Carroll and Snowling, 2001). Since both paradigms assess children’s ability to detect the rhyming phenomenon, some researchers also refer to this ability as “rhyme awareness.”

Syllable segmentation skills also appear at around 4 years of age (Liberman et al., 1974), while phoneme awareness develops later and partly as a consequence of learning to read and write (Scarborough et al., 1998; Goswami, 2002). Liberman et al. (1974) used a tapping task to assess syllable and phoneme segmentation skills in children and found that 46% of four-year-old children could segment syllables but none could segment phonemes. In the study, 90% of six-year-old children were successful with syllable segmentation and 70% were able to segment phonemes. Taken together, these results support the notion of a large-to-small developmental trajectory of phonological awareness (i.e., from large units to small units).

As the first acquired phonological awareness skill, rhyme awareness serves as a stepping stone for the further development of a more fine-grained awareness of syllables and phonemes within a word. Extensive empirical evidence from rigorous longitudinal research has established a causal link between children’s phonological awareness skills and literacy development (Stanovich, 1992; Wagner et al., 1997; Adams, 1998; Torgesen et al., 1999). Rhyme awareness was also found to be directly applied during reading in English. For example, a child knowing how to read the word *beak* finds it easier to read analogous words such as *peak*, *bean*, and *leak*. Such process is referred to as “orthographic analogy”, during which children make a prediction about word pronunciation by using the shared spelling sequence between words (Goswami, 1998). Moreover, rime analogies (e.g., using *peak* to infer the pronunciation of *beak*) were found to be easier than onset analogies (e.g., using *beak* for *bean*) when children try to read unfamiliar words (Goswami, 1986). This evidence suggests that being able to identify words that rhyme is helpful to children who are learning to read.

### Contributors to Phonological Awareness

Vocabulary knowledge is viewed as a support system for the development of phonological processing skills in young children. Phonological processing skills have been found to be related to vocabulary size (e.g., Edwards et al., 2004; Munson et al., 2005). Metsala and Walley (1998) have proposed the Lexical Restructuring Hypothesis, suggesting that the growth of vocabulary knowledge propels the holistic-to-segmental reorganization of phonological representation in young children. Under the pressure of a growing vocabulary, children need to differentiate between onsets, rimes, syllables, and eventually phonemes to make more generalizations about the phonological structure of their language (Walley, 1993, 2008; Metsala, 1997).

One line of relevant research has focused on how phonological neighborhood influences children’s phonological awareness performance. Phonological neighborhood is the total number of words differing from a target word by the addition, substitution or deletion of one phoneme in any position (Luce and Pisoni, 1998). For example, the neighbors of *rat* include *brat*, *rot*, and *at*. Targets from dense phonological neighborhoods have more similar words while targets from sparse neighborhoods have fewer similar words. Studies have arrived at different conclusions regarding the impact of phonological neighborhood density on phonological awareness skills. In Metsala (1999), children aged 3–4 years of age demonstrated better phoneme blending performance (e.g., select the pictures that match the word consisting of the sounds/b/…/℧/…/∫/) with words from dense neighborhoods, but this neighborhood density effect was not found in their onset-rime blending task (e.g., point to the picture with/d/…/I∫/in it).

De Cara and Goswami (2003) argued that these inconsistent findings result from the one-phoneme-different definition of phonological neighborhood because young children do not have phoneme-level representations of words before literacy learning. Young children are more sensitive to the onset-rime level of phonological representations. The authors proposed that rhyme neighborhood density, which is the number of words that rhyme with each other (e.g., rat, cat, hat) would influence young children’s rhyme awareness performance. They designed a rhyme oddity task that required children to listen to three words and verbally repeat the odd (i.e., non-rhyming) word (e.g., Which word is the odd one from “peak,” “dot,” “not”?). Words were selected from dense versus sparse rhyme neighborhoods in balanced numbers. Three types of odd words were created by altering the following phonemes in the rhyming words within a trial: a rime change (e.g., sock/rock/*win*), a vowel change (e.g., hat/rat/*neat*) and a coda change (e.g., feed/need/*deal*). Children’s vocabulary sizes were measured by their raw score on the British Picture Vocabulary Scales. Results showed that four- to five-year-old children with larger vocabulary sizes were better at identifying the odd words from dense rhyme neighborhoods than words from sparse rhyme neighborhoods. This performance difference between dense versus sparse rhyme neighborhood was strongest for the coda change trials, followed by the rhyme change trials but absent for the vowel change trials. Children with weaker vocabulary skills did not show effects of either rhyme neighborhood density or its interaction with type of changes.

In a forced choice classification task, Storkel (2002) found that young children make decisions regarding which CVC word sounds alike based on the overlap in the rhyme of the word (dip – sip) for words from dense neighborhoods. For words from sparse neighborhoods, however, the manner feature of the final phoneme of the rhyme mattered in order for children to identify words as sounding alike (tug-mud). Children’s segmental representation of words from dense neighborhoods is more fine-grained therefore, because they are organized by individual phonemes. Representations from sparse neighborhoods, however, are coarser since children perceive phonemes belonging to the same manner category as sounding the same.

### Factors Influencing Phonological Awareness in Deaf and Hard of Hearing Children, and Those With Cochlear Implants

For deaf and hard of hearing (DHH) children, literacy is a difficult area and their average outcomes are below those of hearing children (Marschark and Spencer, 2010). One possible explanation for this poor outcome lies in the development of DHH children’s phonological awareness. According to Locke’s theory of neurolinguistic development (Locke, 1997), holistic utterances accrued between the fifth to seventh month of young children’s lives form a foundation for analytical reconstruction and the acquisition of phonology, morphology and grammar from 20 to 37 months. Absent or degraded auditory input in DHH children compromises this process and may cause these children to treat the incoming speech signal in larger chucks, such as syllables rather than in phonemes (Briscoe et al., 2001). Indeed, DHH children have been found to have poor performance in tasks assessing rhyme awareness and phoneme awareness (Hanson and Fowler, 1987; Campbell and Wright, 1988, 1990; Harris and Beech, 1998; Sterne and Goswami, 2000).

Recent development in cochlear implant (CI) technology has offered a potential opportunity for profoundly deaf children to receive early auditory input, and achieve better literacy outcomes (Geers, 2003; Lyxell et al., 2008). Individual differences such as age of implantation and working memory have also been investigated in terms of their influence on CI children’s literacy and pre-literacy skills. Yet only a limited number of studies have explored whether CI improve DHH children’s phonological awareness.

A series of recent studies have been conducted by Nittrouer et al. (2012) and colleagues focusing on language and literacy outcomes in children with CI. In the first study, 50 children who had participated in an earlier study between the ages of 12 to 48 months participated at the end of their kindergarten year. The group consisted of children with CI, children with hearing loss wearing hearing aids, and children with normal hearing (NH). Outcome measure was a comprehensive measure combining language comprehension, expressive vocabulary, phonological awareness, literacy skills, narrative skills and speed of processing. Results showed that language comprehension before the age of 24 months was the best predictor for later success. Other strong predictors after the age of 36 months, were vocabulary skills and syntactic complexity (Nittrouer et al., 2012).

In a subsequent study (Nittrouer et al., 2014), the investigators used language samples collected from kindergarteners to investigate how children with CI and children with NH differ in terms of grammatical skills in spontaneous production during personal narratives. Measures of phonological awareness and lexical knowledge were also included. Results showed that children with CI performed at one standard deviation below the control group on language measures, including lexical skills, but two standard deviations below on measures of phonological awareness. Lexical knowledge accounted for variance on three measures of language. One measure of phonological awareness, sensitivity to word-final phonemic structure, as well as number of bound morphemes accounted for variance above and beyond lexical knowledge. No factors related to hearing loss or intervention, except age at first implant, explained variance on language measures. The authors concluded by recommending intervention explicitly supporting grammatical skills for children with CI.

Morphosyntactic and phonological structure appeared to be mutually independent in second graders with NH, but not in children with CI according to results from Nittrouer et al. (2016). The authors found that the development of sensitivity to early predictors for phonological performance in children with CI included auditory comprehension and MLU. Predictors for morphosyntactic skills included MLU and expressive vocabulary. Children with CI were also followed up in 6th grade in Nittrouer et al. (2018). Phonological, lexical and morphosyntactic abilities were measured. It was found that compared to children with NH, deficits remained fairly consistent since earlier studies. The main area of concern was phonological skills, followed by lexical and morphosyntactic skills. Lexical skills and phonological awareness skills developed from second to sixth grade in both children with CI and NH. There were, however, no correlations between phonological awareness and expressive vocabulary at the later point in time, which can probably be explained by the fact that there was a strong correlation between word reading skills and phonological awareness. According to Hogan et al. (2005) phonological awareness and word reading are so strongly correlated at 2nd grade and after, that phonological awareness will not add additional information. It is clear from the studies cited above, however, that phonological awareness remains an area of vulnerability in children with CI.

In a longitudinal study, James et al. (2005) found that 5 to 10-year old children with CI initially had better syllable awareness than rhyme or phoneme awareness and they made significant improvement in their rhyme awareness over a period of 12 months. The authors claimed that the use of CI promotes the advancement of phonological awareness following the syllable – rhyme – phoneme developmental trajectory in TD children with NH. Additionally, the initial phonological awareness of children with CI were compared with a group of profoundly deaf children and another group of severely deaf children, both of which were using hearing aids (HAs) instead of CI. Children with CI were found to have the same level of syllable awareness as the less impaired group with better levels of residual hearing and using HA, but the same level of rhyme awareness skills as the profoundly deaf children using HA. The latter group had similar levels of residual hearing as the children with CI before implantation. The author concluded that cochlear implants benefited DHH children’s syllable awareness, but not rhyme awareness.

In James et al. (2007), two groups of children with CI were recruited. The early group included children implanted between 2 and 3.6 years and the late group children included implanted between 5 and 7 years. Another group of younger reading-matched children with NH also participated. *Z*-scores were calculated for the performance of children with NH performance in a number of phonological awareness tasks. Phonological awareness performance of the early group fell on the lower end of NH children’s *z*-score distribution, while late-implanted children’s scores fell mostly below the distribution. The early group also achieved greater progress over time than the late group overall. Notably, some late-implanted children demonstrated the most improvement. In Johnson and Goswami (2010), early-implanted children (i.e., before the age of three) were also found to have equivalent rhyme awareness performance compared to reading-level matched peers with NH, while late-implanted children (i.e., later than 43 months) had significantly lower performance. When they combined children with CI who performed above chance level from both the early and late groups, they found that these children’s performances were not significantly different from that of their reading matched peers. This suggests that time of implantation is not the only decisive factor. The fact that age of implantation is not the only factor that matters for positive outcomes has also been illustrated in a study by Willstedt-Svensson et al. (2004). These authors found that the best predictor of lexical and grammatical development in children with CI was the percentage correctly imitated vowels in a non-word repetition task, instead of age of implantation. Other factors that are important for a positive outcome are length and quality of intervention, as well as interaction style of parents (Nittrouer, 2010). Overall, these studies suggest that a CI does offer a better chance for DHH children to acquire typical phonological awareness skills. Early implantation is generally more beneficial, but individual outcomes are highly variable.

Another line of research, has investigated the association between verbal working memory, short-term phonological memory (STPM), and the development of language skills in children (Gathercole and Baddeley, 1993). Typically, working memory (WM) tasks are thought to involve both maintenance of information and some type of manipulation simultaneously, which is also the case in phonological awareness tasks. STPM on the contrary, is considered a subskill of WM and only involves rote memory span, such as in a forward digit span task (Kronenberger et al., 2013). It has been shown in a multitude of studies of children with CI that verbal working memory skills, typically measured by digit span tests, is an area of vulnerability (Pisoni and Cleary, 2003; Pisoni et al., 2011; Kronenberger et al., 2013). AuBuchon et al. (2015) showed that even when digit spans are presented visually, WM performance in CI users is lower than that of individuals with typical hearing. The authors suggested that this population experience WM weaknesses that go beyond issues related to audibility and speech production. They provided an explanation that stresses the importance of auditory input for the development of phonological representations in long-term memory, which supports reactivation and recovery in a short-term memory task.

Researchers have used a non-word repetition task and a non-word discrimination task as an index of STPM in children with CI. Non-word repetition is traditionally used to assess the function of the phonological loop in the Baddeley and Gathercole model of working memory (Baddeley, 1986; Gathercole and Baddeley, 1990a). There is a large body of research demonstrating a link between non-word repetition skills and language abilities in children (e.g., Gathercole and Baddeley, 1990a,b; Montgomery, 1995; Sahlén et al., 1999a,b). Some researchers have also used the Competing Language Processing Task (CLPT, Gaulin and Campbell, 1994) to assess WM skills in a dual-processing task. Ibertsson et al. (2009) found that children and adolescents aged 11 to 19, who were CI users, performed poorer on non-word repetition and non-word discrimination compared to the results of NH children aged 5, 7, and 10 pulled from other studies. The CI group’s performance was similar to that of the 14- to 15-year-olds with NH on the WM task, which includes dual processing. Willstedt-Svensson et al. (2004) used non-word repetition, non-word discrimination and an adapted version of the CLPT (Towse et al., 1998) to study STPM, WM, as well as novel word learning in fifteen children 5 to 11 years old with CI devices. Children were congenitally deaf and had received their implants between the age of 2 and 6 years of age. Findings indicated that age of implantation was linked to performance in a novel word learning task. There was also a correlation between performance in the non-word repetition task and the WM task with novel word learning ability. In a paper presenting an overview of studies focusing on cognitive development and communication skills in Swedish-speaking children with CI. Lyxell et al. (2008) found that in tasks requiring phonological processing, CI users typically perform at lower levels than individuals with NH. In other WM tasks, however, the difference between groups is not as prominent, and sometimes even absent. CI user performance on non-verbal WM tasks was investigated by Cleary et al. (2001). These investigators created a WM task requiring memory for sequences of visual-spatial cues or the same cues paired with auditory signals. Children with CI and NH were asked to reproduce each sequence by pressing buttons on a response box. Results showed that the CI users obtained shorter spans on both tasks than the NH children. The children with CI also showed a smaller gain with the addition of auditory cues compared to the NH group. The authors concluded that the results indicate atypical WM development regardless of input modality. This study indicates that auditory deprivation during the first years of life may affect areas above and beyond language, such as WM.

Orthographic information is yet another factor influencing children’s performance in phonological awareness tasks. “Orthographic congruency” describes whether or not the phonological information and the orthographic information of words lead to the same phonological judgment. For example, Campbell and Wright (1988) compared rhyme awareness in DHH children and children with NH. Children were shown pictures of “d*og*/fr*og*” (i.e., congruent) and “h*air*/b*ear*” (i.e., incongruent). In congruent trials, the rimes of the words were spelled and pronounced the same while in incongruent trials, they were spelled differently. Results showed that both children with NH had higher accuracy with congruent trials while DHH children only made correct rhyme judgments with the congruent trials. Research on syllable awareness (Sterne and Goswami, 2000) and phoneme awareness (Miller, 1997) have also found a similar effect of orthographic congruency. Taken together, these studies show that children rely on orthographic information in phonological awareness tasks, but DHH children rely on such information to a larger degree.

The relationship between vocabulary, phonological neighborhood density and phonological awareness in children with CI is less studied. Dillon et al. (2012) found a possible relationship between larger vocabulary size and more robust phonological representations in children with CI. It is unknown if rhyme awareness in children who were implanted early is subject to a rhyme neighborhood density effect and if performance is linked to vocabulary. Children with CI do not tend to reach the same level of vocabulary development as children with NH (Yoshinaga-Itano et al., 2010). Some research has shown that children implanted by the age of 2 have a better chance of achieving receptive vocabulary skills within normal range, however (Hayes et al., 2009). Kirk et al. (1995) found that children with CI are sensitive to phonological neighborhood density in speech recognition the same way as children with NH are. Therefore, it is possible that CI children have the same sensitivity to rhyme neighborhood density as NH children in phonological awareness tasks. However, weaker vocabulary skills may take a toll on CI children’s development of phonological awareness skills.

Assessments of phonological awareness in children with CI could be skewed for three reasons. First, assessment tools fail to recognize that some English phonemes are harder to identify than others, even for people with NH (Cutler et al., 2004). This fact denies fair assessment for children with CI, who may receive auditory input with poorer quality than children with NH. Carroll and Snowling (2001) found that phonologically similar non-rhyming words were the most difficult for children with NH to reject in a rhyme matching task. It is reasonable to assume that children with CI would be even more confused with phonologically similar items. Secondly, when making phonological judgments, DDH children rely more on orthographic transparency (e.g., Sterne and Goswami, 2000), but assessment tools typically do not take this into account. Finally, most assessment tools do not include words from balanced phonological neighborhoods. Meanwhile children with normal NH were found to perform better with words from dense phonological neighborhoods in a phoneme blending task (Metsala, 1999) and in a rhyme oddity task (De Cara and Goswami, 2003).

### Aims of the Current Study and Hypotheses

It is known that general oral language skills matter for the development of phonological awareness skills (Cooper et al., 2002), but in this study we focused on the importance of vocabulary skills for success in a rhyme recognition task. We use a rhyme recognition task (i.e., oddity task), with items created to only contain sound changes with maximal differences in terms of perceptual saliency (Cutler et al., 2004), from dense and sparse rhyme neighborhoods and controlled for orthographic congruency. The study was guided by the following questions:

1. Do *individual differences* in vocabulary knowledge and working memory capacity predict children’s performance on a rhyme recognition task?

We predict that children with better vocabulary knowledge and stronger working memory capacity will perform better in a rhyme recognition task. This prediction is based on past evidence of positive correlations between children’s rhyme awareness skills and vocabulary size or working memory capacity.

2. How do *linguistic characteristics* of words (i.e., rhyme neighborhood density, orthographic congruency and type of sound changes) influence children’s performance in a rhyme oddity task?

Based on De Cara and Goswami (2003), we anticipate that only children with larger vocabulary size will be influenced by rhyme neighborhood density, such that their accuracy will be higher for words from dense rhyme neighborhoods. We also predict that the performance of children with larger vocabulary size will be mediated by the trial types. In coda change trials, children’s accuracy for words from dense rhyme neighborhoods would be significantly higher than words from sparse rhyme neighborhoods. Such differences will not be as prominent in vowel change or rhyme change trials. Children with smaller vocabulary sizes will not show effects of either rhyme neighborhood density or its interaction with type of changes.

Additionally, we expect that children will perform better on orthographically congruent trials than incongruent trials. This prediction is based on past findings that both children with NH and CI rely on orthographic information when making rhyme judgments.

## Materials and Methods

### Participants

Fifteen children with NH (mean age = 5; 2, SD = 10 months) and six congenitally deaf children (mean age = 6; 10, SD = 6 months) with cochlear implants participated in the study. Participants were recruited through distribution of flyers at medical centers, university clinics and public spaces (e.g., libraries, cafés, etc.). Written informed consent was obtained from the parents of all participating children in the study. All the children’s primary language was English. Two children with CI were bilaterally implanted and the other four were unilaterally implanted and used a hearing aid on the contralateral ear. All children with CI were implanted before the age of two. Demographic information of all children is listed in Tables 1, 2.

**TABLE 1:** Demographic information for all participating children.

	**NH**	**CI**
	**median (IQR)**	**median (IQR)**
Chronological age (year; month)	5; 5 (11 months)	6; 11.5 (3.75 months)
PTONI (standard score)	121 (28)	119.5 (20.5)
PPVT (standard score)	121.5 (11.75)	84.5 (4)
PPVT (raw score)	114.5 (34.25)	89 (7.5)
Block recall^1^	4 (1)	4 (0.75)

*^1^Span scores on the block recall in Working Memory Test Battery for Children (WMTB-C).*

**TABLE 2:** Demographic information for children with CI.

**ID**	**Age**	**Sex**	**SES^1^**	**Age at identification (ms)**	**Age at HA fitting (ms)**	**intervention**	**Communication mode**	**Etiology**	**School environment**	**Age at implant (Type of CI processor)**		**Initial stimulation (ms)**	**Aided PTA^4^**
										**I^2^**	**II^3^**		
1	6;10	F	MA	1.5	6	EI	Auditory oral	Unknown, genetics negative	Mainstreamed in Kindergarten, prior School for deaf	0;9 (Cochlear N5)		unknown	16 dB
2	6;10	F	MA	1.5	6	EI	Auditory oral	Unknown, genetics negative	Mainstreamed in Kindergarten, prior School for deaf	0;9 (Cochlear N5)		unknown	16 dB
3	7;2	F	BA	1.5	3	EI	Auditory oral	Unknown	Mainstreamed school	1;0 (Cochlear N5)	4;11 (Cochlear N6)	14	15 dB
4	7;4	F	BA	18	11	Speech-language	Auditory oral	Parents reported Kawasaki disease	School for Deaf	1;9 (Cochlear N5)		unknown	10 dB
5	5;10	M	MA	3	5.5	EI	Total communication	Genetics	School for Deaf	0;10 (Cochlear N5)	1;3 (Cochlear N5)	18	20 dB
6	7;1	M	N/A	4	4	Speech-language	Auditory oral	Unknown	School for Deaf	0;9 (Cochlear N6)		unknown	26 dB

*^1^SES (maternal education). ^2,3^“I” and “II” indicate first and second implantation (if applicable). ^4^Aided PTA (most recently measured prior to the test session).*

### Procedure

Children completed four standardized tests and a rhyme oddity task in a random order to avoid an effect of fatigue on results. Vocabulary was assessed by the Peabody Picture Vocabulary Test – 4 (PPVT-4, Dunn and Dunn, 2007). Children were asked to point to a picture, from a selection of four, that represented the word the experimenter spoke. Non-verbal intelligence was assessed by the Primary Test of Non-verbal Intelligence (PTONI, Ehrler and McGhee, 2008). This task required children to select a picture that did not belong to a set, in terms of visual patterns, by pointing. General language ability was assessed with the Test of Early Language Development, fourth edition (TELD-4, Hresko et al., 2017) for all except one child, who was given the Clinical Evaluation of Language Fundamentals – Preschool 2 (CELF-Preschool 2, Semel et al., 2004). Working memory was measured by the block recall subtest in the Working Memory Test Battery for Children (WMTB-C, Pickering and Gathercole, 2001), which is a non-verbal task where the child points to series of blocks following the sequence modeled by the experimenter. Children with CI completed the experimental procedure in the same way as children with NH, without any adaptation.

### The Rhyme Oddity Task

To assess rhyme awareness, a rhyme oddity task adapted by De Cara and Goswami (2003) was used. The task consisted of 36 trials of three words: two words rhyming with each other, and one word not rhyming with the other two. The non-rhyming word’s position in each trial was semi-randomized, which resulted in six different semi-randomized versions of the task. Each child received one version of the task, with the 36 trials presented in a fully randomized order.

Children saw a picture of a boy looking and listening attentively, which prompted the beginning of each trial. Then an icon of a loudspeaker appeared on the computer screen, while the audio of the first word was played simultaneously. This was then followed by a second speaker icon and the second word; and the third speaker icon and the final word with previous speakers remaining on the screen. Children were instructed to point to the loudspeaker that played the “non-rhyming” word at the end of each trial.

Prior to the experimental trials, a training session was provided. The children first played a rhyming game where the experimenter presented three printed pictures of objects (e.g., star, *egg*, car). Children were asked to point to the non-rhyming picture after the experimenter named the three pictures. After demonstrating an understanding of the task, children moved on to “play this game on the computer.” The computerized task began with six practice trials. In the first two practice trials, the experimenter paused and explained the procedure in a step-by-step manner (e.g., “Do you see the little boy? We need to really listen now! First you will see a speaker and it will play a word. … Can you point to the word that does not rhyme with the other two?”). Children who understood and followed the first two practice trials completed the next four practice trials independently and proceeded to the experiment. Children who had problems with the rhyming game or practice trials were able to repeat any part of the training until they fully understood.

The stimuli words were recorded by a native female speaker of American English using a professional digital recorder (i.e., Fostex FR-2LE). The sound file was edited and normalized in the Audacity software for computer presentation. The stimuli were presented to the children from a laptop computer (i.e., Thinkpad X230) and through a loudspeaker (i.e., Mackie MR mk3) with a Behringer U-control UCA222 soundcard. The stimuli were presented at a 22.05 kHz sampling rate and 65 dB SPL. The speaker was positioned approximately 1 m in front of the children at 0° azimuth.

### Stimuli

Words in the rhyme oddity task were well-controlled for phonological similarity. The stimuli in the rhyme oddity task were single-syllable words with an initial consonant (i.e., onset), a middle vowel (i.e., vowel) and a final consonant (i.e., coda). The vowel and the coda form the rime of words. The perceptual qualities of the vowels, codas, or the rimes in the non-rhyming words were created to be maximally different from their counterparts in the rhyming words by using confusion matrices in Cutler et al. (2004). The confusion matrices provide information about the likelihood of mistaking an English vowel or consonant for another one in background noise by listeners with typical hearing (e.g., confusing/p/for/b/). In the current study, the vowels, codas, and the rimes in the non-rhyming words were the least likely to be confused with those in the rhyming words. In past research, none of the rhyme oddity tasks or rhyme matching tasks using auditory stimuli have taken into consideration the perceptual similarities between speech sounds. It is possible that any performance differences between words from dense versus sparse rhyme neighborhoods may have been affected by the lack of control of perceptual similarities in the rhyming items. The current study circumvents this problem by including stimuli that are as perceptually different as possible.

Three linguistic characteristics of the stimuli words were manipulated in the rhyme oddity task. First, words were selected from both dense and sparse rhyme neighborhoods using the auditory database reported in De Cara and Goswami (2002). Eighteen trials have words from dense rhyme neighborhoods (hereafter dense trials) and the other 18 words from sparse neighborhoods (hereafter sparse trials). A t-test validated that the dense versus sparse manipulation was significant. The mean rhyme neighborhood density for the dense stimuli was 25.3 (SD = 4.0) and the mean rhyme neighborhood density for the sparse stimuli was 7.7 (SD = 2.9), *t*(53) = 25.89, *p* < 0.001.

Additionally, three types of non-rhyming words were created by altering the following phonemes in the rhyming words within a trial: a “rime change” (e.g., sock/rock/*win*), a “vowel change” (e.g., hat/rat/*neat*) and a “coda change” (e.g., feed/need/*deal*).

Finally, orthographic congruency of the stimuli was also controlled by having the rimes (VC2) in half of the rhyming words spelled congruently (e.g., feed/need) and the other half spelled incongruently (e.g., date/wait). Children did not see the spellings of the stimuli, rather, they needed to listen and select the non-rhyming word based on auditory input. These manipulations were made to reveal if children with CI and NH are influenced by orthographic information when making rhyme judgments in an auditory mode.

Word familiarity and age of acquisition were also controlled for in the stimuli. The familiarity ratings of all words were above 6.75 on a 1 to 7 scale as reported in Luce and Pisoni (1998). The age of acquisition ratings are below age 4; 22 using a 1–7 scale (Ages 0–2 = 1, 2–4 = 2, above 13 = 7) (Cortese and Khanna, 2008). Stimuli words and summary statistics for the variables of interest are shown in the Supplementary Appendix A.

### Statistical Analysis

We first investigated whether group differences existed between children’s age, hearing experience, language and cognitive abilities. One NH child did not return for their second session, resulting in missing data in the PPVT and block recall tests. Therefore, this child’s data was not included in the group comparison tests for these two scores. For children with CI, their hearing experience was quantified by their length of amplification use with CI.^1^ For children with NH, experience receiving postnatal auditory input, equals their chronological age. PPVT raw score was used as a proxy for children’s “absolute vocabulary size,” which is common practice in past literature investigating the relationship between phonological processing and vocabulary development (e.g., Gathercole et al., 1991; Metsala, 1999). Wilcoxon rank sum tests were used for group comparisons on children’s chronological age, hearing experience, PPVT raw score and standard score, general language standard score, PTONI standard score and block recall raw score.

Participants received binary scoring for the rhyme oddity task. To answer the first research question concerning the relationship between individual differences and rhyme awareness, a generalized mixed-effect logistic regression was fitted to this binary outcome variable using the lme4 package (Bates et al., 2015) in RStudio Version 1.0.136 (R Development Core Team, 2017) and following Harel and McAllister (2019). The fixed effect structure included the following predictor variables: PPVT raw score, Block recall span score, Group (NH versus CI), and interactions between Group and all the other variables. All predictor variables except for group were transformed into *z*-scores to facilitate model convergence. The Group variable was sum-coded to allow for interpretation of other variables as overall predictors of accuracy performance. The random effects included test items and participant.

To answer the second research question concerning the association between linguistic characteristics and children’s rhyme awareness, a second mixed-effect logistic regression was fitted to participants’ binary accuracy data. The fixed effect structure included the following predictor variables: Group, PPVT_r, RND, Ortho, Change, two-way interactions between Group and PPVT_r, PPVT_r and Change, PPVT_r and RND, as well as a three-way interaction term between PPVT_r, RND and Change. Again, Group, RND, Ortho and Change were sum-coded to allow for interpretation of other variables as overall predictors of accuracy performance. The random effects included test items and participant.

## Results

### Group Comparison

Results from the Wilcoxon rank sum tests (Table 3) revealed that NH children’s chronological age was significantly lower than that of the children with CI (*z* = −2.56, *p* = 0.01), but that the group of CI children’s time with CI amplification was similar to the chronological age of the NH children (*z* = −1.14, *p* = 0.13). NH children had significantly higher language scores (*z* = −3.04, *p* < 0.001) and vocabulary scores (PPVT raw scores *z* = −1.85, *p* = 0.03) compared with children with CI. However, there were no group differences on any of the non-language related measures including non-verbal intelligence (PTONI, *z* = −0.46 *p* = 0.32) or working memory capacity (Block Recall, *z* = 0.24, *p* = 0.59).

**TABLE 3:** Wilcoxon rank sum tests results comparing NH and CI on their age, hearing age and standardized tests scores.

		***n***	***z*-value**	***p***
Age	NH	15	–2.56	*p* = 0.01^∗^
	CI	6		
Hearing experience	NH	15	–1.14	*p* = 0.13
	CI	6		
PPVT_s^1^	NH	14	–3.24	*p* < 0.001^∗^
	CI	6		
PPVT_r^2^	NH	14	–1.85	*p* = 0.03^∗^
	CI	6		
Language^3^ (standard score)	NH	15	–3.04	*p* < 0.001^∗^
	CI	6		
PTONI (standard score)	NH	15	–0.46	*p* = 0.32
	CI	6		
Block recall	NH	14	0.24	*p* = 0.59
	CI	6		

*^1,2^PPVT_s: PPVT standard score derived from chronological age; PPVT_r: PPVT raw score. ^3^One child completed CELF for language assessment while all other children completed TELD.*

### Individual Differences

**FIGURE 1:**
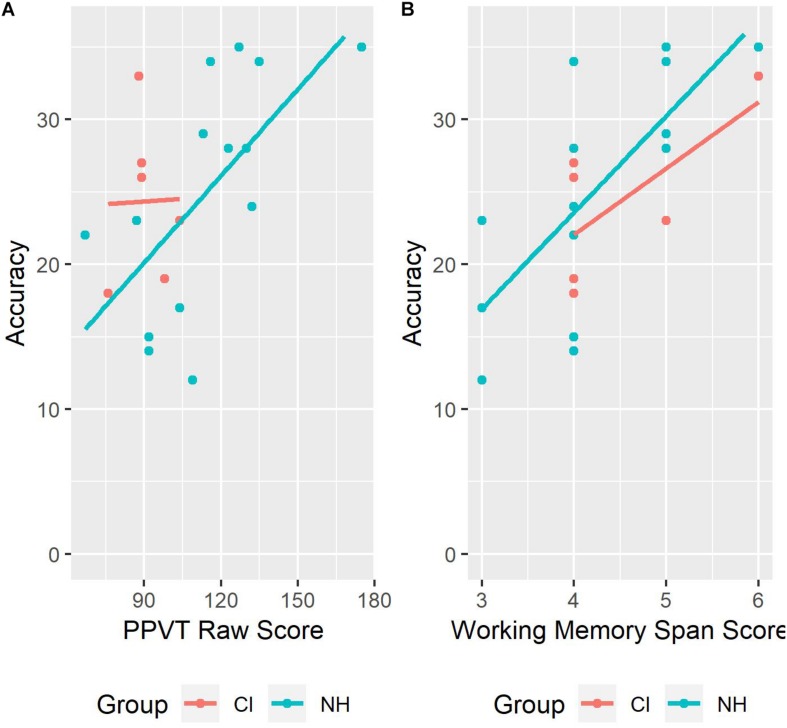
Rhyme oddity task performance as a function of vocabulary **(A)** and working memory **(B)**.

Spearman’s correlations of predictor variables are summarized in Table 4. Correlations are shown without a Bonferroni correction, since this procedure is overly conservative according to Perneger (1998). Results from our first model (Table 5) showed significant effects of group (β = −0.36, *p* < 0.001) suggesting that children with CI had lower average performance than children with NH at the rhyme awareness task. The association between PPVT_r and rhyme awareness was significant (β = 0.05, *p* < 0.001), with a positive slope indicating that, on average, children with larger vocabulary size were more successful at the task. The interaction between group and vocabulary was significant (β = −0.47, *p* < 0.001), suggesting that the slopes for vocabulary were different between children with NH and CI, as can be seen in Figure 1A. The association between WM and rhyme awareness was significant (β = 0.82, *p* < 0.001) with a positive slope suggesting that children with better WM skills had better rhyme awareness performance. The interaction between group and WM was also significant (β = −0.06, *p* < 0.001), suggesting that the slopes for WM were different between children with NH and CI, as can be seen in Figure 1B.

**TABLE 4:** Spearman’s correlation matrix for independent variables.

	**1**	**2**	**3**	**4**	**5**	**6**
Chron. Age (*n* = 21)	–					
PPVT_s (*n* = 20)	–0.39^∗∗∗^	–				
PPVT_r (*n* = 20)	0.05	0.83^∗∗∗^	–			
General language (*n* = 21)	–0.35^∗∗∗^	0.81^∗∗∗^	0.59^∗∗∗^	–		
Block recall span (*n* = 20)	0.46^∗∗∗^	0.12^∗∗^	0.31^∗∗∗^	0.19^∗∗∗^	–	
PTONI (*n* = 21)	0.16^∗∗∗^	0.26^∗∗∗^	0.35^∗∗∗^	0.58^∗∗∗^	0.26^∗∗∗^	–

*^∗^*p* < 0.05, ^∗∗^*p* < 0.01, ^∗∗∗^*p* < 0.001.*

**TABLE 5:** Regression results for individual differences.

	**Estimate**	**Std. error**	**Statistic**	***p* value**
(Intercept)	0.77	0.00	393.42	*p* < 0.001
Group^1^	–0.36	0.00	–189.02	*p* < 0.001
PPVT_r^2^	0.05	0.00	25.38	*p* < 0.001
WM^3^	0.82	0.00	429.41	*p* < 0.001
Group × PPVT_r	–0.47	0.00	–244.78	*p* < 0.001
Group × WM	–0.06	0.00	–32.53	*p* < 0.001
(Random effect) item	0.59			
(Random effect) subject	0.45			

*^1^Group: CI (cochlear implant) or NH (normal hearing). ^2^PPVT_r, Peabody Picture Vocabulary Test raw score. ^3^WM, working memory.*

To probe these two interactions, we divided the children based on groups (NH versus CI) and performed two additional models on these two groups, respectively. In Table 6, results for NH children show that the association between PPVT_r and rhyme awareness was significant (β = 0.53, *p* < 0.05) with a positive slope suggesting that NH children with larger vocabulary size were more successful at the task. The association between WM and rhyme awareness was also significant (β = 0.90, *p* < 0.001), with a positive slop suggesting that NH children with better working memory skills had better rhyme awareness performance. In Table 7, results showed that the association between PPVT_r and rhyme awareness was not significant in the CI group. The association between WM and rhyme awareness was significant in the CI group (β = 0.68, *p* < 0.05) with a positive slope suggesting that CI children with better WM skills had better rhyme awareness performance.

**TABLE 6:** Regression results for individual difference in the NH group.

	**Estimate**	**Std. error**	**Statistic**	***p* value**
(Intercept)	1.19	0.20	5.91	*p* < 0.001
PPVT_r	0.53	0.23	2.27	*p* < 0.05
WM	0.90	0.23	3.96	*p* < 0.001
(Random effect) item	0.54			
(Random effect) Subject	0.48			

**TABLE 7:** Regression results for individual differences in the CI group.

	**Estimate**	**Std. error**	**Statistic**	***p* value**
(Intercept)	0.94	0.27	3.48	*p* < 0.001
PPVT_r	−0.11	0.24	−0.44	*p* = 0.66
WM	0.68	0.29	2.35	*p* < 0.05
(Random effect) item	0.77			
(Random effect) Subject	0.41			

### Characteristics of Items in the Rhyme Recognition Task

As illustrated in Table 8, results from our second mixed-effects logistic model did not show a significant effect for Group, PPVT_r, Change or Ortho. There was no significant interaction between Group and PPVT_r, PPVT_r and RND, PPVT_r and Change and no significant three-way interaction between PPVT_r, RND and Change.

**TABLE 8:** Regression results for linguistic characteristics.

	**Estimate**	**Std. error**	**Statistic**	***p* value**
(Intercept)	0.89	0.43	2.07	*p* = 0.04
Group	–0.03	0.43	–0.06	*p* = 0.95
PPVT_r	0.50	0.54	0.92	*p* = 0.36
RND^1^	–0.12	0.11	–1.06	*p* = 0.29
Change1^2^	0.03	0.16	0.22	*p* = 0.83
Change2	–0.25	0.15	–1.66	*p* = 0.10
Ortho^3^	0.00	0.10	–0.03	*p* = 0.97
Group × PPVT_r	–0.51	0.54	–0.95	*p* = 0.34
PPVT_r × RND	0.00	0.12	–0.04	*p* = 0.97
PPVT_r × Change1	0.18	0.17	1.04	*p* = 0.30
PPVT_r × Change2	–0.20	0.16	–1.27	*p* = 0.20
PPVT_r × RND × Change1	0.06	0.16	0.39	*p* = 0.70
PPVT_r × RND × Change2	–0.14	0.15	–0.92	*p* = 0.36
(Random effect) item	0.54			
(Random effect) subject	0.85			

*^1^RND, rhyme neighborhood density. ^2^ Change: type of changes in the rime-ending of the non-rhyming words. ^3^Ortho, orthographic congruency.*

### Qualitative Analyses of Vocabulary Size, Rhyme Awareness and Linguistic Characteristics

**FIGURE 2:**
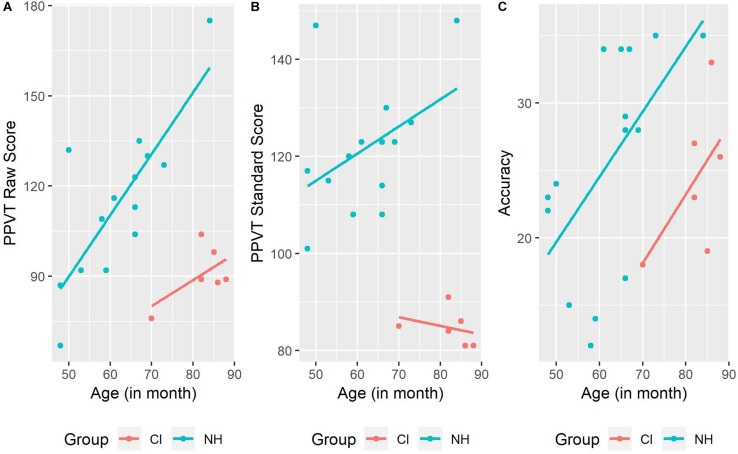
Relationship between chronological age and PPVT raw score **(A)**, PPVT standard score **(B)**, and accuracy performance in the rhyme oddity task **(C)**.

We conducted two additional descriptive analyses to qualitatively explore the relationship between vocabulary size, rhyme awareness and linguistic characteristics. In the first analysis, we plotted bivariate relationships between three pairs of variables: PPVT raw score and chronological age; PPVT standard score and chronological age; rhyme awareness performance and chronological age (Figures 2A–C). Figure 2A shows a pattern of increasing PPVT raw scores in NH children with increasing chronological age. This pattern was still present for the NH children when PPVT scores were reported as standard scores (Figure 2B). There are only six children with CI and therefore no clear conclusions can be made, but the same pattern does not seem to be present in this small group during visual inspection (Figures 2A,B). Both CI and NH children appeared to perform better in the rhyme awareness task with increasing age based on visual inspection of the graphs (Figure 2C).

**FIGURE 3:**
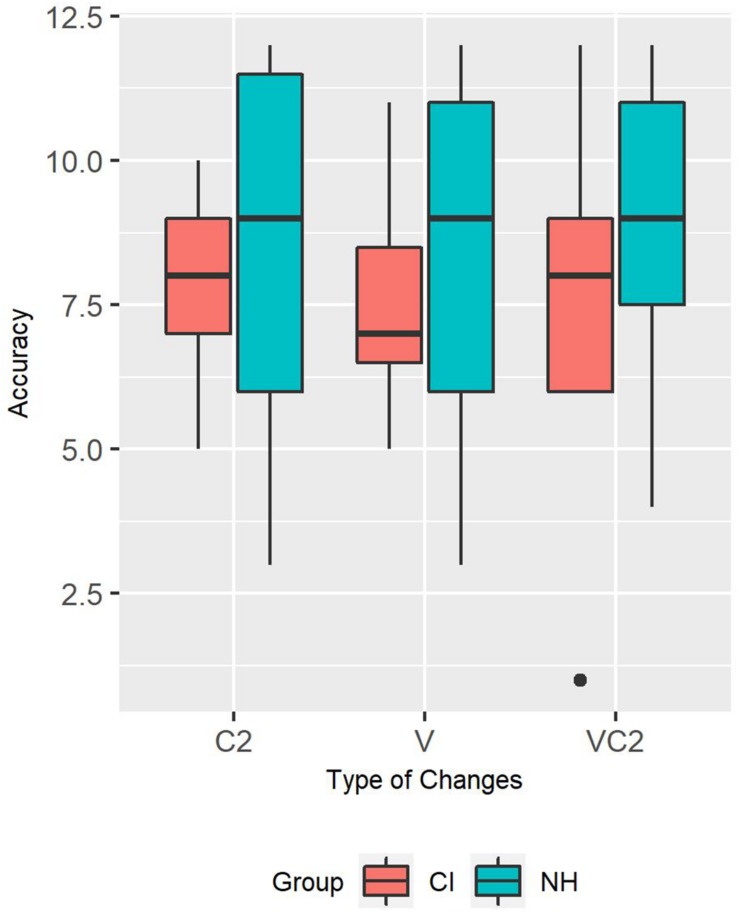
Accuracy for trials with a C2, V, and VC2 change (by group).

The second analysis was a qualitative exploration of which type of non-rhyming words were the most challenging for children with NH and CI, respectively. NH and CI children performance on the trials containing non-rhyming words with a C2, V, and VC changes were plotted in Figure 3. Visual qualitative inspection revealed that children with NH performed similarly with the three types of non-rhyming words. Children with CI seemed to be slightly more challenged when the non-rhyming word differed from the rhyming word by a change in the middle vowel (V-change).

## Discussion

In this study we explored how vocabulary skills and working memory matter for phonological awareness skills in children. We included a small group of six congenitally deaf children with CI, who had been implanted before the age of two. Compared to many previous studies, which have included children with a wide range of age of implantation, our group all children had been implanted early. The children with CI were older than the NH children, but had similar hearing experience and non-verbal intelligence. In the rhyme recognition task, we intentionally maximized the difference of perceptual saliency of words within each trial to grant fair assessment of rhyme awareness in children with CI. Making sure that the non-rhyming word in each trial has a minimal probability of perceptual confusion with the rhyming words is of particular importance when assessing phonological processing in children with hearing impairments. Poorer success rates compared to children with NH may otherwise not be a function of poorer phonological processing skills but may be secondary to less optimal auditory input.

Our results show that vocabulary size measured by PPVT raw scores, predicted success in the rhyme awareness task among children with NH. Other studies have found that phonological processing skills are related to vocabulary size (e.g., Edwards et al., 2004; Munson et al., 2005). In Metsala (1999), performance on phonological awareness tasks was related to overall vocabulary size, age of acquisition of words, and neighborhood density. Researchers have shown that vocabulary skills are important for the development of phonological awareness skills and have suggested that the holistic to segmental development of phonological awareness skills is a secondary effect of vocabulary acquisition. As a child learns more words, there is a need to make distinctions between increasingly smaller segments because many words have dense phonological neighborhoods (Metsala and Walley, 1998). The children with CI in our study had poorer vocabulary skills compared with the NH children, which is consistent with previous research showing that vocabulary skills develop slower in children with CI (Yoshinaga-Itano et al., 2010). We did not find a positive correlation between vocabulary size and rhyme awareness in our children with CI. This finding is in contrast with the results from Dillon et al. (2012) who found that in children with CI vocabulary size was a mediating factor between reading skills and phonological awareness skills. In their study, there was a weaker correlation between phonological awareness and reading when vocabulary was controlled. Figures 2A,B in our study, show that one CI child was slightly younger than the remaining five, and had a lower PPVT raw score. In the older five children with CI, the PPVT standard score had a negative slope, indicating that the vocabulary skills of these children might not have developed following a predicted pattern over time. There was a positive correlation between accuracy rates in the rhyme awareness task and chronological age, however, which might indicate that other factors were more important in supporting these children in developing their phonological awareness skills. Since our study has a small sample size of children with CI, we remain cautious in interpreting these results.

Contrary to our expectation, we did not find a significant interaction between rhyme neighborhood density and vocabulary size, as measured by PPVT raw score. Children with larger vocabulary sizes performed comparably with words from dense versus sparse neighborhoods and so did children with smaller vocabulary sizes. One explanation may be that our version of the rhyme oddity task is less taxing compared to the earlier version in De Cara and Goswami (2003), since we intentionally minimized the perceptual similarity between trial words. Storkel (2002) found that children had more detailed segmental representation of words from dense neighborhoods than words from sparse neighborhoods. Consequently, children found it more difficult to judge whether words sound the same when these words were from sparse neighborhoods. In words from sparse neighborhoods, children perceived words ending with sounds from the same category in terms of manner of articulation as the same (tug-mud). However, since our stimuli from sparse neighborhoods were made to be maximally different from each other, this might have reduced the level of difficulty while children made decisions about rhyming. This may be a reason why children showed similar performance with words from dense versus sparse neighborhoods.

We were not able to replicate the three-way interaction between vocabulary size, rhyme neighborhood density and type of changes reported in De Cara and Goswami (2003). Our results indicated that children’s performance was equally accurate in the coda change, vowel change, and rime change trials and no rhyme neighborhood effects were shown in any type of changes. This null finding is, however, consistent with some earlier studies, in which no performance differences were found between coda change conditions and vowel change conditions (Bradley and Bryant, 1983; Kirtley et al., 1989; Bryant et al., 1990). One explanation provided by De Cara and Goswami (2003) for their novel finding is that their rhyme oddity task with 5-year-olds used pre-recorded speech stimuli. The auditory nature of the stimuli did not provide lip cues. Therefore, children could only rely on linguistic cues to make rhyme judgments. Since a coda change trial provide the least number of linguistic cues (i.e., a consonant) compared to the vowel and the rhyme change trials, it is the most linguistically demanding condition and might be the most discriminative condition for detecting an effect of rhyme neighborhood density. Our rhyme oddity task was reduced in terms of perceptual similarity between trial words, however. This might have caused a loss of discriminating power in the coda trials, and thus suppressed rhyme neighborhood density effects. As can be seen during visual inspection of Figure 3, our children with CI seemed to be most challenged by rhyme changes including a vowel change. Perhaps CI children tend to rely on acoustic information carried in the vowel when processing speech, which made this sound change particularly difficult in spite of the fact that we had made changes as salient as possible.

Many of the participating children were old enough to have been exposed to orthographic forms in reading and may have stored not only phonological forms of words, but also orthographic forms. It is not well known how orthographic representations support individuals in phonological processing tasks, although we know that orthographic support facilitates word learning in children with developmental language disorders (Ricketts et al., 2015). Our results revealed no significant effects for orthographic congruency, however. Past studies that have identified such effect have either used written tasks, or a picture identification task without any auditory stimuli (e.g., Campbell and Wright, 1988; Miller, 1997; Sterne and Goswami, 2000). In written tasks, readily available information of orthographic congruency would have a direct impact on children’s rhyme judgments. In picture identification tasks, children must access the phonological information of the words through lexical retrieval, which may activate of the words’ orthographies. Children in our study only heard the pronunciation of the stimulus words and might have processed and analyzed the phonological components of these words without activating their orthographic representation. As a result, orthographic congruency did not show an influence on children’s performance in the rhyme oddity task.

Non-verbal working memory skills were not different between children with NH and children with CI with similar hearing experience. On the surface level, this result contradicts the results from Cleary et al. (2001), where children with CI performed worse than children with NH on tasks assessing non-verbal working memory. However, a closer look revealed that the CI children in their study had shorter hearing experience than the chronologically age-matched children with NH. Correlation coefficients in the current study (Table 4) also showed that working memory scores had a stronger correlation with hearing experience than with chronological age. Together, this suggests that hearing experience contributes to working memory skills in children with CI. Our finding that non-verbal working memory predicts children’s rhyme awareness is consistent with previous findings that phonological processing skills are linked to children’s short-term memory skills regardless of hearing status (Pisoni and Geers, 2000; Pisoni and Cleary, 2003; Willstedt-Svensson et al., 2004).

To summarize, we found that both vocabulary size and non-verbal working memory skills are important factors for rhyme recognition skills in children with NH. In children with CI, only working memory was found to be significant. However, vocabulary learning is still important for children with CI. The children with CI in our study had poorer vocabulary skills than children with NH. Past research (Dillon et al., 2012) has found a positive relationship between vocabulary and children’s phonological awareness skills. Nittrouer et al. (2018) did not find a strong correlation between expressive vocabulary and phonological awareness in 6th grade children with NH or with CI, however. Our study has a very limited sample size of children with CI, and therefore results are difficult to generalize. For our NH children, the results indicate a positive relationship between vocabulary skills and rhyme awareness, which is consistent with earlier studies on children with NH (e.g., Metsala and Walley, 1998; Edwards et al., 2004; Munson et al., 2005). Finally, working memory skills are important for phonological awareness tasks regardless of hearing status. This finding is expected based on previous literature, and also suggests that mentally comparing items in a phonological awareness task involves a memory component.

The current study is a first attempt to use a rhyme recognition task with a stringent control of perceptual similarity of distinguishing phonemes, which might have reduced the level of difficulty in task. Increasing the level of saliency of the distinguishing phonemes in the task may have had an effect on how rhyme neighborhood density or type of rhyme changes in our task played a role. This may also be a reason why we did not find an effect of orthographic congruency. Future studies might examine whether different levels of perceptual similarities of stimuli would have an effect on children’s performance in rhyme awareness tasks. Such studies may also lead to the development of balanced stimuli to be included in standardized rhyme awareness tests. Task administration was randomized. Randomization may, however, have affected the robustness of the correlations. The most important limitation of the current study is the small number of children in the CI group. The small sample size also makes it difficult to investigate the impact of background characteristics and other factors, such as parental engagement on children’s rhyme awareness skills. In future studies the goal will be to include a more balanced number of participants in the groups to study phonological processing skills in this population.

## Data Availability

The datasets generated for this study are available on request to the corresponding author.

## Ethics Statement

This study was carried out in accordance with the recommendations of the New York University Committee on Activities Involving Human Subjects, the Institutional Review Board of Northwell Health, and the NYU Langone Medical School Office of Science and Research Institutional Review Board with written informed consent from all subjects’ caregivers. All subjects’ caregivers were given written informed consent in accordance with the Declaration of Helsinki. The protocol was approved by the New York University Committee on Activities Involving Human Subjects, the Institutional Review Board of Northwell Health, and the NYU Langone Medical School Office of Science and Research Institutional Review Board.

## Author Contributions

LJ designed the study, collected the data, analyzed the results, and wrote the manuscript. KV contributed to the design of the study through her expertise in audiology, and provided input regarding the analyses. AM collected the data from children with CI and scored standardized tests. CR participated in all aspects of the project except the data collection.

## Conflict of Interest Statement

The authors declare that the research was conducted in the absence of any commercial or financial relationships that could be construed as a potential conflict of interest.

## Footnotes

1 Some CI children received amplification through hearing aids before their CI implantation, yet the auditory benefit of their hearing aids was deemed inadequate which is why they qualified to be eligible for CIs.

## References

[B1] Adams M. J. (1998). *Beginning to Read: Thinking and Learning About Print (10. Print).* Cambridge, MA: MIT Press.

[B2] AuBuchon A. M. Pisoni D. B. Kronenberger W. G. (2015). Short-term and working memory impairments in early-implanted, long-term cochlear implant users are independent of audibility and speech production. *Ear Hear.* 36 733–737. 10.1097/AUD.0000000000000189 26496666 PMC4621773

[B3] Baddeley A. D. (1986). *Working Memory. Oxford Psychology Series, 11.* Oxford: Clarendon Press.

[B4] Bates D. Mächler M. Bolker B. Walker S. (2015). Fitting linear mixed-effects models using lme4. *J. Stat. Softw.* 67 1–48. 10.18637/jss.v067.i01

[B5] Bradley L. Bryant P. E. (1983). Categorizing sounds and learning to read-a causal connection. *Nature* 301 419–421. 10.1038/301419a0

[B6] Briscoe J. Bishop D. V. M. Frazier Norbury C. (2001). Phonological processing, language, and literacy: a comparison of children with mild-to-moderate sensorineural hearing loss and those with specific language impairment. *J. Child Psychol. Psychiatr.* 42 329–340. 10.1017/S0021963001007041 11321202

[B7] Bryant P. E. MacLean M. Bradley L. L. Crossland J. (1990). Rhyme and alliteration, phoneme detection, and learning to read. *Dev. Psychol.* 26 429–438. 10.1037/0012-1649.26.3.429

[B8] Campbell R. Wright H. (1988). Deafness, spelling and rhyme: how spelling supports written word and picture rhyming skills in deaf subjects. *Q. J. Exp. Psychol. A* 40 771–788. 10.1080/14640748808402298 3212212

[B9] Campbell R. Wright H. (1990). Deafness and immediate memory for pictures: dissociations between “inner speech” and the “inner ear”? *J. Exp. Child Psychol.* 50 259–286. 10.1016/0022-0965(90)90042-7 2258691

[B10] Carroll J. M. Snowling M. J. (2001). The effects of global similarity between stimuli on children’s judgment of rime and alliteration. *Appl. Psycholinguist.* 22 327–342. 10.1017/S0142716401003034 17113063

[B11] Cleary M. Pisoni D. Geers A. (2001). Some measures of verbal and spatial working memory in eight- and nine-year-old hearing-impaired children with cochlear implants. *Ear Hear.* 22 395–411. 10.1097/00003446-200110000-00004 11605947 PMC3429119

[B12] Cooper D. H. Roth F. P. Speece D. L. Schatschneider C. (2002). The contribution of oral language skills to the development of phonological awareness. *Appl. Psycholinguist.* 23 399–416. 10.1017/S0142716402003053

[B13] Cortese M. J. Khanna M. M. (2008). Age of acquisition ratings for 3,000 monosyllabic words. *Behav. Res. Methods* 40 791–794. 10.3758/BRM.40.3.791 18697675

[B14] Cutler A. Weber A. Smits R. Cooper N. (2004). Patterns of English phoneme confusions by native and non-native listeners. *J. Acoust. Soc. Am.* 116 3668–3678. 10.1121/1.1810292 15658717

[B15] De Cara B. Goswami U. (2002). Similarity relations among spoken words: the special status of rimes in English. *Behav. Res. Methods Instrum. Comput.* 34 416–423. 10.3758/bf03195470 12395558

[B16] De Cara B. Goswami U. (2003). Phonological neighbourhood density: effects in a rhyme awareness task in five-year-old children. *J. Child Lang.* 30 695–710. 10.1017/S0305000903005725 14513474

[B17] Dillon C. M. de Jong K. Pisoni D. B. (2012). Phonological awareness, reading skills, and vocabulary knowledge in children who use cochlear implants. *J. Deaf Stud. Deaf Educ.* 17 205–226. 10.1093/deafed/enr043 22057983 PMC3598411

[B18] Dunn L. M. Dunn D. M. (2007). *PPVT-4: Peabody Picture Vocabulary Test.* Upper Saddle River, NJ: Pearson.

[B19] Edwards J. Beckman M. Benjamin M. (2004). The interaction between vocabulary size and phonotactic probability effects on children’s production accuracy and fluency in nonword repetition. *J. Speech Lang. Hear. Res.* 47 421–436. 10.1044/1092-4388(2004/034) 15157141

[B20] Ehrler D. J. McGhee R. L. (2008). *PTONI: Primary Test of Nonverbal Intelligence.* Austin, TX: Pro-Ed.

[B21] Gathercole S. E. Baddeley A. D. (1990a). Phonological memory deficits in language disordered children: is there a causal connection? *J. Mem. Lang.* 29 336–360. 10.1016/0749-596X(90)90004-J

[B22] Gathercole S. E. Baddeley A. D. (1990b). The role of phonological memory in vocabulary acquisition: a study of young children learning new names. *Br. J. Psychol.* 81 439–454. 10.1111/j.2044-8295.1990.tb02371.x

[B23] Gathercole S. E. Baddeley A. D. (1993). Phonological working memory: a critical building block for reading development and vocabulary acquisition? *Eur. J. Psychol. Educ.* 8:259 10.1007/BF03174081

[B24] Gathercole S. E. Willis C. Baddeley A. D. (1991). Differentiating phonological memory and awareness of rhyme: reading and vocabulary development in children. *Br. J. Psychol.* 82 387–406. 10.1111/j.2044-8295.1991.tb02407.x

[B25] Gaulin C. A. Campbell T. F. (1994). Procedure for assessing verbal working memory in normal school-age children: some preliminary data. *Percept. Mot. Skills* 79 55–64. 10.2466/pms.1994.79.1.55 7991333

[B26] Geers A. E. (2003). Predictors of reading skill development in children with early cochlear implantation. *Ear Hear.* 24(Suppl.), 59S–68S. 10.1097/01.AUD.0000051690.43989.5D 12612481

[B27] Goswami U. (1986). Children’s use of analogy in learning to read: a developmental study. *J. Exp. Child Psychol.* 42 73–83. 10.1016/0022-0965(86)90016-0

[B28] Goswami U. (1998). “The role of analogies in the development of word recognition,” in *Word Recognition in Beginning Literacy*, eds Metsala J. L. Ehri L. C. (Mahwah, NJ: Lawrence Erlbaum Associates Publishers).

[B29] Goswami U. (2002). Phonology, reading development, and dyslexia: a cross-linguistic perspective. *Ann. Dyslexia* 52 139–163. 10.1007/s11881-002-0010-0

[B30] Hanson V. L. Fowler C. A. (1987). Phonological coding in word reading: evidence from hearing and deaf readers. *Mem. Cognit.* 15 199–207. 10.3758/BF03197717 3600259

[B31] Harel D. McAllister T. (2019). Multilevel models for communication sciences and disorders. *J. Speech Lang. Hear. Res.* 62 783–801. 10.1044/2018_JSLHR-S-18-0075 30969889

[B32] Harris M. Beech J. R. (1998). Implicit phonological awareness and early reading development in prelingually deaf children. *J. Deaf Stud. Deaf Educ.* 3 205–216. 10.1093/oxfordjournals.deafed.a014351 15579864

[B33] Hayes H. Geers A. E. Treiman R. Moog J. S. (2009). Receptive vocabulary development in deaf children with cochlear implants: achievement in an intensive auditory-oral educational setting. *Ear Hear.* 30 128–135. 10.1097/AUD.0b013e3181926524 19125035

[B34] Hogan T. P. Catts H. W. Little T. D. (2005). The relationship between phonological awareness and reading. *Lang. Speech Hear. Serv. Sch.* 36 285–293. 16389701 10.1044/0161-1461(2005/029) PMC2848754

[B35] Hresko W. P. Reid D. K. Hammill D. D. (2017). *Test of early language development: TELD-4.* Austin, TX: Pro-ed, Inc.

[B36] Ibertsson T. Hansson K. Asker-Àrnason L. Sahlén B. (2009). Speech recognition, working memory and conversation in children with cochlear implants. *Deafness Educ. Int.* 11 132–151. 10.1002/dei.261

[B37] James D. Rajput K. Brinton J. Goswami U. (2007). Phonological awareness, vocabulary, and word reading in children who use cochlear implants: does age of implantation explain individual variability in performance outcomes and growth? *J. Deaf Stud. Deaf Educ.* 13 117–137. 10.1093/deafed/enm042 17728276

[B38] James D. Rajput K. Brown T. Sirimanna T. Brinton J. Goswami U. (2005). Phonological awareness in deaf children who use cochlear implants. *J. Speech Lang. Hear. Res.* 48 1511–1528. 10.1044/1092-4388(2005/105) 16478387

[B39] Johnson C. Goswami U. (2010). Phonological awareness, vocabulary, and reading in deaf children with cochlear implants. *J. Speech Lang. Hear. Res.* 53 237–261. 10.1044/1092-4388(2009/08-0139) 20008682

[B40] Kirk K. I. Pisoni D. B. Osberger M. J. (1995). Lexical effects on spoken word recognition by pediatric cochlear implant users. *Ear Hear.* 16 470–481. 10.1097/00003446-199510000-00004 8654902 PMC3495322

[B41] Kirtley C. Bryant P. MacLean M. Bradley L. (1989). Rhyme, rime, and the onset of reading. *J. Exp. Child Psychol.* 48 224–245. 10.1016/0022-0965(89)90004-0 2794855

[B42] Kronenberger W. G. Pisoni D. B. Harris M. S. Hoen H. M. Xu H. Miyamoto R. T. (2013). Profiles of verbal working memory growth predict speech and language development in children with cochlear implants. *J. Speech Lang. Hear. Res.* 56 805–825. 10.1044/1092-4388(2012/11-0356) 23275401 PMC3700625

[B43] Liberman I. Y. (1973). 1. Segmentation of the spoken word and reading acquisition. *Bull. Orton Soc.* 23 64–77. 10.1007/BF02653842 10660904

[B44] Liberman I. Y. Shankweiler D. Fischer F. W. Carter B. (1974). Explicit syllable and phoneme segmentation in the young child. *J. Exp. Child Psychol.* 18 201–212. 10.1016/0022-0965(74)90101-5 23303378

[B45] Locke J. L. (1997). A theory of neurolinguistic development. *Brain Lang.* 58 265–326. 10.1006/brln.1997.1791 9182750

[B46] Luce P. A. Pisoni D. B. (1998). Recognizing spoken words: the neighborhood activation model. *Ear Hear.* 19 1–36. 10.1097/00003446-199802000-00001 9504270 PMC3467695

[B47] Lundberg I. Frost J. Petersen O.-P. (1988). Effects of an extensive program for stimulating phonological awareness in preschool children. *Read. Res. Q.* 23 263–284. 10.1598/rrq.23.3.1

[B48] Lyxell B. Sahlén B. Wass M. Ibertsson T. Larsby B. Hällgren M. (2008). Cognitive development in children with cochlear implants: relations to reading and communication. *Int. J. Audiol.* 47(Suppl. 2), S47–S52. 10.1080/14992020802307370 19012112

[B49] Marschark M. Spencer P. E. (2010). Promises (?) of deaf education: from research to practice and back again. *Oxford Handbook Deaf Stud. Lang. Educ.* 2 1–4.

[B50] Metsala J. L. (1997). An examination of word frequency and neighborhood density in the development of spoken-word recognition. *Mem. Cogn.* 25 47–56. 10.3758/bf03197284 9046869

[B51] Metsala J. L. (1999). Young children’s phonological awareness and nonword repetition as a function of vocabulary development. *J. Educ. Psychol.* 91 3–19. 10.1037/0022-0663.91.1.3

[B52] Metsala J. L. Walley A. C. (1998). “Spoken vocabulary growth and the segmental restructuring of lexical representations: precursors to phonemic awareness and early reading ability,” in *Word Recognition in Beginning Literacy*, eds Metsala J. L. Ehri L. C. (Mahwah, NJ: Erlbaum).

[B53] Miller P. (1997). The effect of communication mode on the development of phonemic awareness in prelingually deaf students. *J. Speech Lang. Hear. Res.* 40 1151–1163. 10.1044/jslhr.4005.1151 9328886

[B54] Montgomery J. W. (1995). Sentence comprehension in children with specific language impairment: the role of phonological working memory. *J. Speech Hear. Res.* 38 187–199. 10.1044/jshr.3801.187 7731209

[B55] Munson B. Swenson C. Manthei S. (2005). Lexical and phonological organization in children. *J. Speech Lang. Hear. Res.* 48 108–124. 10.1044/1092-4388(2005/009) 15938063

[B56] Nittrouer S. (2010). *Early Development of Children with Hearing Loss.* San Diego, CA: Plural Publishing.

[B57] Nittrouer S. Caldwell A. Holloman C. (2012). Measuring what matters: effectively predicting language and literacy in children with cochlear implants. *Int. J. Pediatr. Otorhinolaryngol.* 76 1148–1158. 10.1016/j.ijporl.2012.04.024 22648088 PMC3383903

[B58] Nittrouer S. Lowenstein J. H. Holloman C. (2016). Early predictors of phonological and morphosyntactic skills in second graders with cochlear implants. *Res. Dev. Disabil.* 55 143–160. 10.1016/j.ridd.2016.03.020 27078086 PMC4961612

[B59] Nittrouer S. Muir M. Tietgens K. Moberly A. C. Lowenstein J. H. (2018). Development of phonological, lexical, and syntactic abilities in children with cochlear implants across the elementary grades. *J. Speech Lang. Hear. Res.* 61 2561–2577. 10.1044/2018_JSLHR-H-18-0047 30242344 PMC6428240

[B60] Nittrouer S. Sansom E. Low K. Rice C. Caldwell-Tarr A. (2014). Language structures used by kindergartners with cochlear implants: relationship to phonological awareness, lexical knowledge and hearing loss. *Ear Hear.* 35 506–518. 10.1097/AUD.0000000000000051 24992492 PMC4142107

[B61] Perneger T. V. (1998). What’s wrong with bonferroni adjustments. *BMJ* 316 1236–1238. 10.1136/bmj.316.7139.1236 9553006 PMC1112991

[B62] Pickering S. Gathercole S. (2001). *Working Memory Test Battery for Children (WMTB-C): Manual.* London: Pearson.

[B63] Pisoni D. Cleary M. (2003). Measures of working memory span and verbal rehearsal speed in deaf children after cochlear implantation. *Ear Hear.* 24 106S–120S. 10.1097/01.AUD.0000051692.05140.8E 12612485 PMC3434463

[B64] Pisoni D. Geers A. (2000). Working memory in deaf children with cochlear implants: correlations between digit span and measures of spoken language processing. *Ann. Otol., Rhinol. Laryngol.* 185 92–93. 10.1177/0003489400109s1240 PMC3429114 11141023

[B65] Pisoni D. Kronenberger W. Roman A. Geers A. (2011). Article 7: measures of digit span and verbal rehearsal speed in deaf children following more than 10 years of cochlear implantation. *Ear Hear.* 32 60S–74S. 10.1097/AUD.0b013e3181ffd58e 21832890 PMC3080130

[B66] R Development Core Team (2017). *R: A Language and Environment for Statistical Computing.* Vienna: R Foundation for Statistical Computing.

[B67] Ricketts J. Dockrell J. E. Patel N. Charman T. Lindsay G. (2015). Do children with specific language impairment and autism spectrum disorders benefit from the presence of orthography when learning new spoken words? *J. Exp. Child Psychol.* 134 43–61. 10.1016/j.jecp.2015.01.015 25795987

[B68] Sahlén B. Reuterskiold-Wagner C. Nettelbladt U. Radeborg K. (1999a). Non-word repetition in children with language impairment: pitfalls and possibilities. *Int. J. Lang. Commun. Disord.* 34 337–352. 10.1080/136828299247441 10884905

[B69] Sahlén B. Wagner C. R. Nettelbladt U. Radeborg K. (1999b). Language comprehension and non-word repetition in children with language impairment. *Clin. Linguist. Phon.* 13 369–380. 10.1080/026992099299031

[B70] Scarborough H. S. Ehri L. C. Olson R. K. Fowler A. E. (1998). The fate of phonemic awareness beyond the elementary school years. *Sci. Stud. Read.* 2 115–142. 10.1207/s1532799xssr0202_2

[B71] Semel E. Wiig E. H. Secord W. A. (2004). *Clinical Evaluation of Language Fundamentals-Preschool-2 (CELF-Preschool-2).* San Antonio, TX: Psychological Corp.

[B72] Stanovich K. E. (1992). “Speculations on the causes and consequences of individual differences in early reading acquisition,” in *Reading Acquisition*, eds Gough P. B. Ehri L. C. Treiman R. (Hillsdale, NJ: Lawrence Erlbaum Associates, Inc).

[B73] Sterne A. Goswami U. (2000). Phonological awareness of syllables, rhymes, and phonemes in deaf children. *J. Child Psychol. Psychiatr.* 41 609–625. 10.1111/1469-7610.00648 10946753

[B74] Storkel H. L. (2002). Restructuring of similarity neighbourhoods in the developing mental lexicon. *J. Child Lang.* 29 251–274. 10.1017/s0305000902005032 12109371

[B75] Torgesen J. K. Wagner R. K. Rashotte C. A. (1994). Longitudinal studies of phonological processing and reading. *J. Learn. Disabil.* 27 276–286. 10.1177/002221949402700503 8006506

[B76] Torgesen J. K. Wagner R. K. Rashotte C. A. Rose E. Lindamood P. Conway T. (1999). Preventing reading failure in young children with phonological processing disabilities: group and individual responses to instruction. *J. Educ. Psychol.* 91 579–593. 10.1037/0022-0663.91.4.579

[B77] Towse J. N. Hitch G. J. Hutton U. (1998). A reevaluation of working memory capacity in children. *J. Mem. Lang.* 39 195–217. 10.1006/jmla.1998.2574

[B78] Wagner R. K. Torgesen J. K. Rashotte C. A. Hecht S. A. Barker T. A. Burgess S. R. (1997). Changing relations between phonological processing abilities and word-level reading as children develop from beginning to skilled readers: a 5-year longitudinal study. *Dev. Psychol.* 33:468. 10.1037/0012-1649.33.3.468 9149925

[B79] Walley A. C. (1993). The role of vocabulary development in childrens spoken word recognition and segmentation ability. *Dev. Rev.* 13 286–350. 10.1006/drev.1993.1015

[B80] Walley A. C. (2008). *In Blackwell Handbooks in Linguistics: Vol. The Handbook of Speech Percpetion.* Malden: Blackwell Publishing.

[B81] Willstedt-Svensson U. Löfqvist A. Almqvist B. Sahlén B. (2004). Is age at implant the only factor that counts? The influence of working memory on lexical and grammatical development in children with cochlear implants. *Int. J. Audiol.* 43 506–515. 10.1080/14992020400050065 15726841

[B82] Yoshinaga-Itano C. Baca R. L. Sedey A. L. (2010). Describing the trajectory of language development in the presence of severe-to-profound hearing loss: a closer look at children with cochlear implants versus hearing aids. *Otol. Neurotol.* 31 1268–1274. 10.1097/MAO.0b013e3181f1ce07 20818291 PMC3014847

[B83] Ziegler J. C. Goswami U. (2005). Reading acquisition, developmental dyslexia, and skilled reading across languages: a psycholinguistic grain size theory. *Psychol. Bull.* 131 3–29. 10.1037/0033-2909.131.1.3 15631549

