# Supplementary material for: Rhyme Awareness in Children With Normal Hearing and Children With Cochlear Implants: An Exploratory Study
Source: Front Psychol. 2019 Sep 12;10:2072. doi: 10.3389/fpsyg.2019.02072 (PMC6751305; doi:10.3389/fpsyg.2019.02072)
Supplement: Supplementary file 1 [file Table_1.DOCX]

**Appendix A. Stimuli**

| **Dense RND** | | **Rhyming Word 1** | | | | | **Rhyming Word 2** | | | | | **Non-rhyming Word** | | | | |
| --- | --- | --- | --- | --- | --- | --- | --- | --- | --- | --- | --- | --- | --- | --- | --- | --- |
| **O** | **Cha** |  | **RND** | **ND** | **Fam.** | **AoA** |  | **RND** | **ND** | **Fam.** | **AoA** |  | **RND** | **ND** | **Fam.** | **AoA** |
| O+ | C2 | **feed** | 22 | 33 | 7.00 | 3.10 | **need** | 22 | 33 | 6.92 | 2.71 | **deal** | 22 | 35 | 7.00 | 3.74 |
| O+ | C2 | **back** | 28 | 53 | 7.00 | 2.65 | **rack** | 28 | 56 | 7.00 | 4.00 | **jam** | 22 | 32 | 6.83 | 3.31 |
| O+ | C2 | **pit** | 24 | 52 | 7.00 | 3.56 | **sit** | 24 | 57 | 7.00 | 2.31 | **chin** | 21 | 31 | 7.00 | 2.59 |
| O- | C2 | **rain** | 38 | 64 | 7.00 | 2.57 | **cane** | 38 | 57 | 7.00 | 3.78 | **gate** | 26 | 46 | 7.00 | 3.34 |
| O- | C2 | **role** | 27 | 53 | 7.00 | 4.22 | **goal** | 27 | 43 | 6.92 | 3.52 | **cone** | 20 | 47 | 7.00 | 3.50 |
| O- | C2 | **date** | 26 | 40 | 7.00 | 3.83 | **wait** | 26 | 57 | 7.00 | 2.88 | **maze** | 22 | 40 | 7.00 | 3.77 |
| O+ | V | **hat** | 24 | 45 | 7.00 | 2.42 | **rat** | 24 | 53 | 7.00 | 2.84 | **neat** | 25 | 47 | 7.00 | 3.41 |
| O+ | V | **kick** | 25 | 48 | 7.00 | 2.97 | **sick** | 25 | 57 | 7.00 | 2.63 | **knock** | 23 | 37 | 7.00 | 3.41 |
| O+ | V | **cheat** | 25 | 33 | 7.00 | 4.10 | **seat** | 25 | 53 | 7.00 | 2.81 | **hot** | 25 | 41 | 7.00 | 2.29 |
| O- | V | **moan** | 20 | 42 | 6.50 | 4.32 | **phone** | 20 | 37 | 7.00 | 2.57 | **jail** | 36 | 45 | 7.00 | 3.41 |
| O- | V | **fight** | 29 | 47 | 7.00 | 3.09 | **kite** | 29 | 42 | 7.00 | 2.91 | **mat** | 24 | 52 | 7.00 | 2.88 |
| O- | V | **sign** | 24 | 40 | 7.00 | 3.38 | **mine** | 24 | 41 | 7.00 | 2.35 | **yawn** | 22 | 34 | 7.00 | 3.09 |
| O+ | VC2 | **sock** | 23 | 42 | 7.00 | 2.38 | **rock** | 23 | 45 | 6.67 | 2.63 | **win** | 21 | 59 | 7.00 | 3.00 |
| O+ | VC2 | **ship** | 26 | 35 | 7.00 | 2.78 | **zip** | 26 | 30 | 7.00 | 3.47 | **ball** | 28 | 55 | 7.00 | 2.16 |
| O+ | VC2 | **tall** | 28 | 46 | 7.00 | 2.84 | **wall** | 28 | 51 | 7.00 | 2.97 | **seek** | 28 | 55 | 6.92 | 4.22 |
| O- | VC2 | **cheek** | 28 | 42 | 7.00 | 2.87 | **beak** | 28 | 50 | 6.25 | 3.63 | **coal** | 27 | 54 | 7.00 | 3.81 |
| O- | VC2 | **yacht** | 25 | 29 | 6.75 | 4.78 | **pot** | 25 | 47 | 7.00 | 3.23 | **will** | 30 | 70 | 7.00 | 2.80 |
| O- | VC2 | **soup** | 20 | 32 | 7.00 | 2.53 | **loop** | 20 | 33 | 6.92 | 3.87 | **rash** | 22 | 39 | 6.58 | 3.66 |

| **Sparse RND** | | **Rhyming Word 1** | | | | | **Rhyming Word 2** | | | | | **Non-rhyming Word** | | | | |
| --- | --- | --- | --- | --- | --- | --- | --- | --- | --- | --- | --- | --- | --- | --- | --- | --- |
| **O** | **Cha** |  | **RND** | **ND** | **Fam.** | **AoA** |  | **RND** | **ND** | **Fam.** | **AoA** |  | **RND** | **ND** | **Fam.** | **AoA** |
| O+ | C2 | **kid** | 9 | 31 | 7.00 | 2.48 | **lid** | 9 | 40 | 7.00 | 3.13 | **wish** | 5 | 35 | 6.92 | 3.03 |
| O+ | C2 | **nose** | 8 | 17 | 7.00 | 2.26 | **rose** | 8 | 31 | 6.83 | 3.00 | **toad** | 11 | 24 | 7.00 | 2.94 |
| O+ | C2 | **toss** | 11 | 22 | 7.00 | 3.35 | **boss** | 11 | 29 | 7.00 | 3.81 | **doll** | 4 | 22 | 6.92 | 2.44 |
| O- | C2 | **should** | 5 | 12 | 7.00 | 3.23 | **good** | 5 | 14 | 7.00 | 2.16 | **push** | 3 | 12 | 6.92 | 2.70 |
| O- | C2 | **full** | 3 | 18 | 7.00 | 2.94 | **wool** | 3 | 20 | 7.00 | 4.03 | **look** | 7 | 19 | 7.00 | 2.53 |
| O- | C2 | **hurt** | 10 | 27 | 7.00 | 2.77 | **shirt** | 10 | 21 | 7.00 | 2.40 | **worm** | 7 | 25 | 7.00 | 2.71 |
| O+ | V | **house** | 6 | 14 | 7.00 | abs | **mouse** | 6 | 19 | 7.00 | abs | **worse** | 7 | 21 | 7.00 | 3.81 |
| O+ | V | **wife** | 5 | 26 | 7.00 | 3.35 | **life** | 5 | 23 | 7.00 | 3.00 | **roof** | 7 | 29 | 7.00 | 3.25 |
| O+ | V | **mud** | 10 | 34 | 7.00 | 2.75 | **bud** | 10 | 45 | 6.83 | 4.00 | **loud** | 5 | 21 | 6.92 | 2.77 |
| O- | V | **third** | 8 | 11 | 6.50 | 2.87 | **word** | 8 | 32 | 7.00 | 2.78 | **hood** | 5 | 15 | 6.75 | 3.56 |
| O- | V | **comb** | 10 | 29 | 7.00 | 3.19 | **foam** | 10 | 20 | 6.92 | 3.47 | **same** | 13 | 30 | 7.00 | 2.83 |
| O- | V | **chief** | 9 | 16 | 7.00 | 3.80 | **beef** | 9 | 23 | 7.00 | 3.38 | **safe** | 2 | 16 | 7.00 | 3.00 |
| O+ | VC2 | **dove** | 5 | 27 | 7.00 | 3.58 | **love** | 5 | 23 | 6.67 | 2.35 | **wipe** | 8 | 29 | 7.00 | 3.22 |
| O+ | VC2 | **kiss** | 8 | 28 | 7.00 | 2.48 | **miss** | 8 | 37 | 7.00 | 2.94 | **pup** | 4 | 26 | 7.00 | 3.06 |
| O+ | VC2 | **like** | 13 | 37 | 7.00 | 2.58 | **hike** | 13 | 28 | 7.00 | 3.75 | **join** | 5 | 17 | 7.00 | 3.59 |
| O- | VC2 | **juice** | 11 | 17 | 7.00 | 2.06 | **goose** | 11 | 19 | 7.00 | 2.77 | **bird** | 8 | 32 | 7.00 | 2.32 |
| O- | VC2 | **hope** | 13 | 30 | 6.92 | 3.59 | **soap** | 13 | 26 | 7.00 | 2.56 | **cage** | 8 | 15 | 7.00 | 3.19 |
| O- | VC2 | **youth** | 5 | 13 | 7.00 | 4.34 | **tooth** | 5 | 13 | 7.00 | 2.28 | **size** | 9 | 18 | 7.00 | 3.06 |

RND = number of rhyme neighbors; ND=number of overall neighbors [both based on the lexical data base reported in De Cara & Goswami (2002)]

O = orthographic congruency; O+ = orthographic congruent; O- = orthographic incongruent

Cha = type of change; C2 = coda change; V = vowel change; VC = rhyme change

Fam. = item familiarity [ranking out of a maximum of 7 according to the Luce & Pisoni’s (1998) adult]

AoA = age of acquisition [ranking from a 7-point scale (1: age 0–2 years; 7: age 13 years and older) from Cortese & Khanna (2008)]

Abs = information absent from the corpus
